# Biomechanical changes of tree shrew posterior sclera during experimental myopia, after retrobulbar vehicle injections, and crosslinking using genipin

**DOI:** 10.1038/s41598-024-71276-8

**Published:** 2024-09-05

**Authors:** Gianfranco Bianco, Christopher A. Girkin, Brian C. Samuels, Massimo A. Fazio, Rafael Grytz

**Affiliations:** 1 Department of Ophthalmology and Visual Sciences, University of Alabama at Birmingham, Birmingham, AL 35294 USA; 2 Department of Biomedical Engineering, University of Alabama at Birmingham, Birmingham, AL 35294 USA

**Keywords:** Medical research, Experimental models of disease, Biomedical engineering

## Abstract

Myopia is a common ocular condition characterized by biomechanical weakening revealed by increasing creep rate, cyclic softening scleral thinning, change of collagen fibril crimping, and excessive elongation of the posterior sclera resulting in blurred vision. Animal studies support scleral crosslinking as a potential treatment for myopia control by strengthening the weakened sclera and slowing scleral expansion. While multiple studies investigated aspects of the biomechanical weakening and strengthening effects in myopia and after scleral crosslinking, a comprehensive analysis of the underlying mechanical changes including the effect of vehicle injections is still missing. The purpose of this study was to provide a comprehensive analysis of biomechanical changes by scleral inflation testing in experimental myopia, after retrobulbar vehicle injections and scleral crosslinking using genipin in tree shrews. Our results suggest that biomechanical weakening in myopia involves an increased creep rate and higher strain levels at which collagen fibers uncrimp. Both weakening effects were reduced after scleral crosslinking using genipin at doses that were effective in slowing myopia progression. Vehicle injections increased mechanical hysteresis and had a small but significant effect on slowing myopia progression. Also, our results support scleral crosslinking as a potential treatment modality that can prevent or counteract scleral weakening effects in myopia. Furthermore, vehicle solutions may cause independent biomechanical effects, which should be considered when developing and evaluating scleral crosslinking procedures.

## Introduction

Myopia or nearsightedness is a common ocular condition with a prevalence of 22.9% and it has been estimated to increase epidemically to 49.8% by 2050 in the worldwide population^1^. While mild (0-1.5 D) and moderate (-1.5D-6.0 D) myopia can be corrected by wearing glasses or contact lenses, high-myopia affecting about 2.7% of myopia cases represents a well-known risk factor for pathological complications^2^ like glaucoma^3,4^, retinal detachment^5^, and macular degeneration^2^. Myopia is characterized by excessive elongation of the back of the eye (posterior sclera), resulting in a progressive shift of the retina away from the plane of focus resulting in blurred vision. Understanding the mechanisms that underlie myopia development is critical in the development of appropriate treatment targets to prevent, slow, or control myopia progression. Several experimental studies using animal models demonstrated that progressive myopia is associated with remodeling of the scleral extracellular matrix likely caused by collagen sliding^6–8^ that leads to a progressive thinning of the sclera and an elongation of the vitreous chamber depth and axial length^9^. From the biomechanical standpoint, the viscoelastic properties of the sclera change during myopia development suggesting a biomechanical weakening of the myopic sclera^10,11^. In the tree shrew model of myopia, the creep rate i.e. the strain rate under a constant load, was found to be significantly increased compared to control eyes^6^ supporting the notion that collagen fibrils are more susceptible to mechanically induced sliding in myopic eyes. Levy et al*.* found that experimental myopia significantly weakens the tree shrew sclera against cyclic loads (cyclic softening)^12^. Another tree shrew study using strip testing data suggested that myopia increases the collagen fiber crimp angle and strain level at which collagen fibers uncrimp within the posterior sclera^13^. The collagen fibers naturally organize in a wavy structure and are assembled into interwoven bundles (lamellae) that straighten under tensile load inducing a fiber recruitment process with a progressive straightening of collagen fibers (uncrimping)^14^. The collagen fiber crimp angle can be interpreted as a metric of the strain level at which crimped fibers transition into straight fibers (uncrimped) under tensile load^15^. In a previous work^13^, the authors found that myopia development changed the crimping response of scleral collagen fibrils, while no changes have been observed in scleral stiffness. The aforementioned studies analyzed the properties of tree shrew sclera by uniaxial tensile tests of strip samples dissected from the sclera, which does not reflect the multiaxial strain experienced in the living condition and may compromise the integrity of the collagen network. As demonstrated in tree shrew^16^ and porcine scleras^17^, uniaxial tests showed a more compliant response and a higher preconditioning effect compared to inflation experiments, which better mimics the physiological loading condition of the intact scleral shell. In fact, the higher compliance response of scleral strips is likely caused by the compromised integrity of the collagen network after sample preparation. To date, only a few animal studies have analyzed the biomechanical change of the myopic scleral shell using inflation testing. Wildsoet et al. tested intact eyes of young chicks by inflation to analyze changes in scleral biomechanics induced by experimental myopia^18^. The authors proposed a load-driven approach to quantify creep-like deformations at a constant pressure of 100 mmHg tracking two-dimensional (2D) displacements at 5 locations across the posterior sclera using one camera. While the approach provided important insight into the biomechanical response of myopic sclera, the results address only one biomechanical effect (creep) and cannot be projected to physiological pressure conditions. In a previous work^16^, we proposed an inflation testing setup, which allows us to extract three-dimensional (3D) data (shape and deformation) to characterize the scleral response. This 3D approach allows us to detect a series of biomechanical effects at both, physiological and high pressure levels^16^. A 3D analysis was also proposed by Campbell et al. to quantify efficacy of collagen cross-linking agents to induce stiffening of rat sclera during inflation testing limiting the study to *ex vivo* experiments^19^.

In order to slow or even halt progressive scleral remodeling and prevent biomechanical weakening in myopia, scleral crosslinking (SXL) has been proposed as potential treatment modality^19–25^. Levy et al. demonstrated that SXL by incubating tree shrew scleral strips in a solution of phosphate-buffer solution (PBS) and 0.25 mM of genipin (a natural crosslinking reagent, extracted from the *Gardenia jasminoides* plant) can reverse the cycling softening response seen in myopic tree shrew sclera^12^. El Hamdaoui et al. evaluated the effect of retrobulbar injections of sham solution or genipin at different concentrations (10 and 20 mM) and numbers of repeated injections (3 and 5 injections), finding that SXL can slow axial elongation and myopia progression in tree shrews^9^. Surprisingly, the authors found that sham injections also had a transient but significant effect on slowing vitreous chamber elongation. Among the known collagen crosslinking agents that do not require light activation, genipin is one of the best characterized and most potent agents. Although alternative crosslinking agents have been shown to be less cytotoxic than genepin at equal concentrations^26^ genepin is more potent and requires a lower concentration to effectively stiffen the sclera^19^ and slow myopia progression^27^. While SXL using ultraviolet light-activated riboflavin can significantly stiffen the sclera as shown in guinea pigs, its application remains challenging as current techniques require the excision of ocular muscles to expose the sclera to the ultraviolet light^23,28,29^.

To the best knowledge of the authors, a comprehensive analysis of the nonlinear elastic and viscoelastic biomechanical changes of the sclera in myopia and after SXL while accounting for the potential effect of the vehicle solution is still missing. The purpose of the present study was to provide a comprehensive assessment of the biomechanical weakening effects induced by experimental myopia, strengthening effects after SXL, and mostly unknown effects of retrobulbar vehicle injections in tree shrews using scleral inflation testing and full-field 3D measurements of scleral shell.

## Results

The results are presented as absolute difference between Right and Left eye (Δ=R-L) for the Normal group, and between Treated and Control eye (Δ=T−C) for groups with monocular experimental myopia. The FD Treated eye was exposed to no injections (FD), three (FD3X) or five repeated retrobulbar genipin injections (FD5X), or five vehicle injections (FDsh). We performed two statistical comparisons at 35 DVE: (i) Normal vs FD group to quantify the effects induced by experimental myopia; (ii) and FD vs FDsh/FD3X/FD5X groups to analyze the effects of SXL and vehicle injections on myopic eyes and to investigate if SXL can reverse myopia-induced biomechanical weakening of the sclera.

**Fig. 1:**
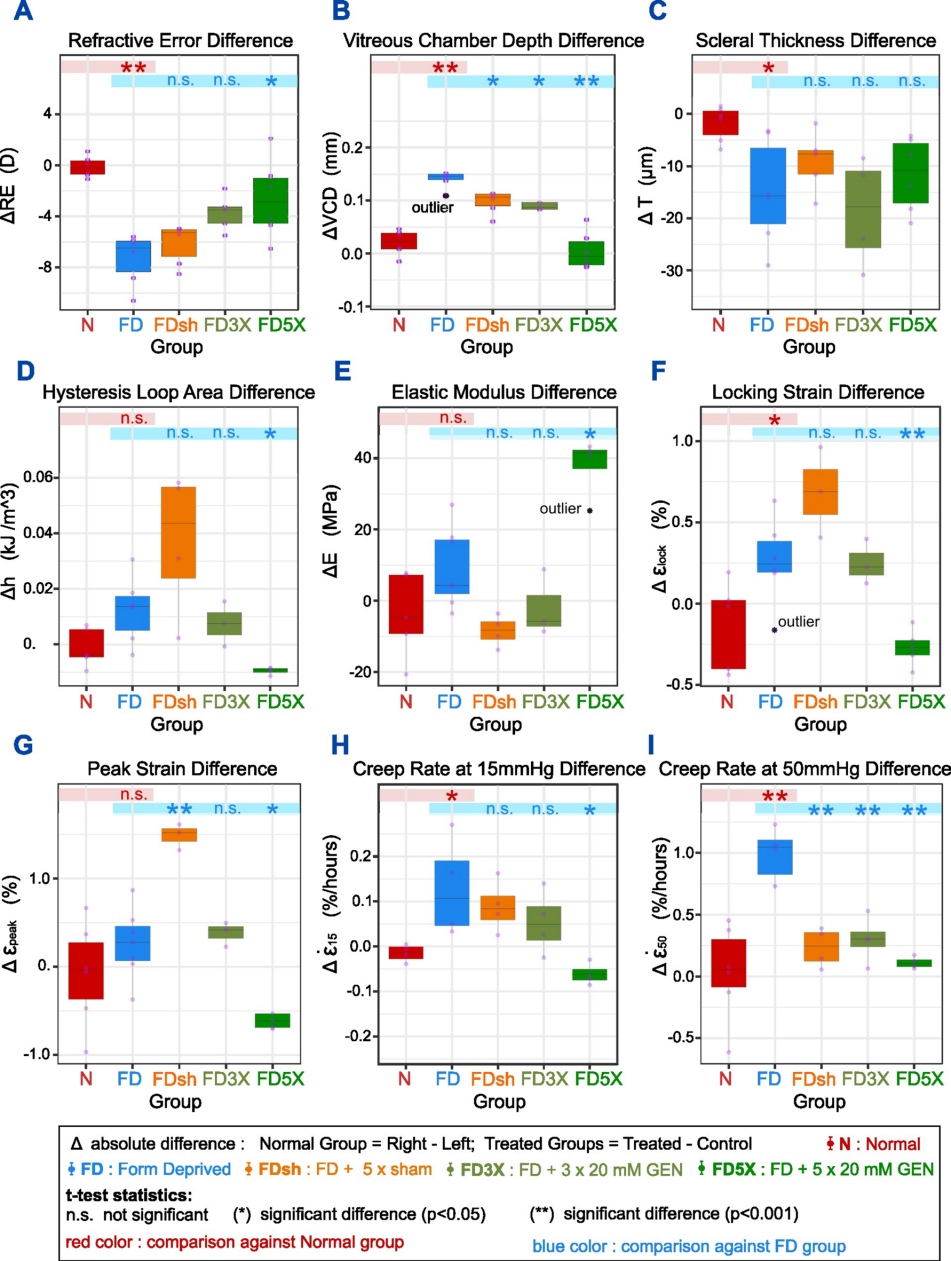
Box plots and statistical results (unpaired *t*-tests) are shown for the differences of refractive error (**a**), vitreous chamber depth (**b**), scleral thickness (**c**), and six biomechanical properties (**d-i**), hysteresis loop area, elastic modulus at high IOPs, locking strain, peak strain, creep rate at 15 and 50 mmHg). Results are reported as absolute differences between Right and Left eyes (Δ = R−L) for the Normal group and between Treated and Control eye (Δ = T−C) for all other groups.

The longitudinal ocular changes from 18 to 35 days of visual experience (DVE) induced by form-deprivation (FD) myopia, vehicle injection and SXL have been presented previously^9,30^. For the reader’s convenience and to demonstrate that FD myopia was effectively induced, we have reported the refraction error (RE), vitreous chamber depth (VCD), and scleral thickness at the experimental endpoint (35 DVE) in Table 1 and Fig. 1.

**Table 1:**
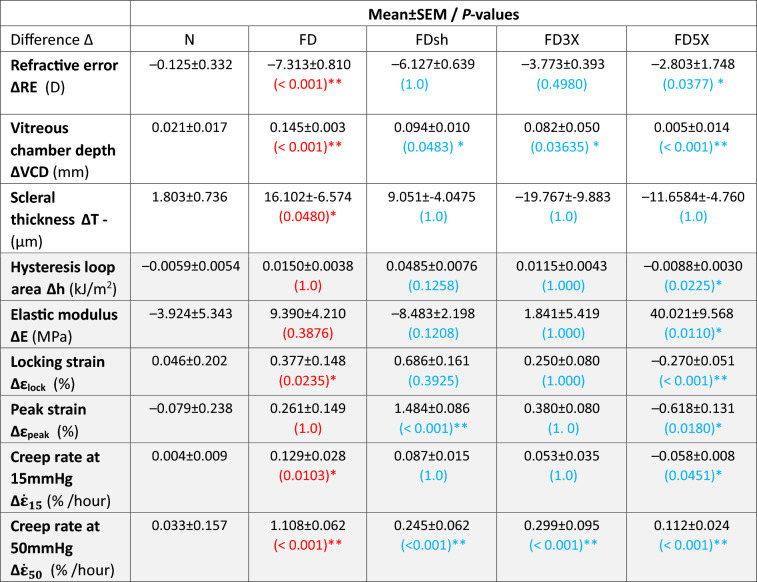
Summary of refractive error, vitreous chamber depth, and scleral thickness differences obtained in vivo at the experimental endpoint (35 DVE) and six biomechanical properties obtained ex vivo.

*p*-values resulting from the comparisons: Normal vs FD group and FD vs FDsh/FD3X/FD5X groups are shown in red and blue font, respectively. The values of the mechanical features are highlighted in gray.

FD myopia caused significant VCD elongation and a myopic RE shift compared to the Normal group. Compared to the FD group, both SXL groups (FD3X, FD5X) and the sham injection group (FDsh) showed a significant decrease in the VCD difference, while only the FD5X showed a significant RE difference. The difference in scleral thickness (ΔT, Table 1) was significantly decreased in the FD group compared to Normal. Interestingly, ΔT of the three groups with retrobulbar injections (FDsh/FD3X/FD5X) remained negative with no significant difference compared to the FD group. While SXL had a clear treatment effect on ΔRE and ΔVCD in the group FD5X, this treatment effect did not manifest in recovering scleral thinning.

### Changes in the biomechanical properties of the sclera

#### Mechanical features of the loading-unloading test

The mechanical response of the posterior sclera was analyzed by studying six mechanical features that were obtained after preconditioning from one load-unload test and two creep tests. Figures 1d–g and Table 1 summarize the results (mean±SEM) and statistical analysis of four biomechanical properties of the sclera calculated from the preconditioned loading-unloading inflation test. The hysteresis loop area difference *Δh* significantly decreased in highest genipin dose group (FD5X) compared to the FD group. Among all treated groups, only the FD5X group showed a negative *Δh* average value, meaning that the hysteresis was lower in the treated eyes as compared with the control eyes. As expected, SXL with 5 genipin injections (FD5X) caused a significant increase in the elastic modulus difference *ΔE* with respect to FD. No significant differences of *ΔE* have been found for all other groups. Compared to the Normal group, the locking strain difference *Δε*_*lock*_ was significantly higher in the FD group compared to Normal. Compared to the FD group, only 5 genipin injections (FD5X) caused a significant decrease in *Δε*_*lock*_. The changes in *Δε*_*lock*_ show a similar trend as seen in the hysteresis loop area *Δh* where the increased locking response during FD myopia was decreased in the highest genipin dose group FD5X but amplified in the sham injection group FDsh. It has to be noted that the locking strain represents the level of strain at which crimped collagen fibril straighten suggesting that sham and genipin injections can have opposite effects on this biomechancial response. Similar to the trend seen in *Δε*_*lock*_, sham injections caused a significant increase in the peak strain difference *Δε*_*peak*_ while 5 genipin injections caused a significant decrease in *Δε*_*peak*_ compared to FD.

**Fig. 2:**
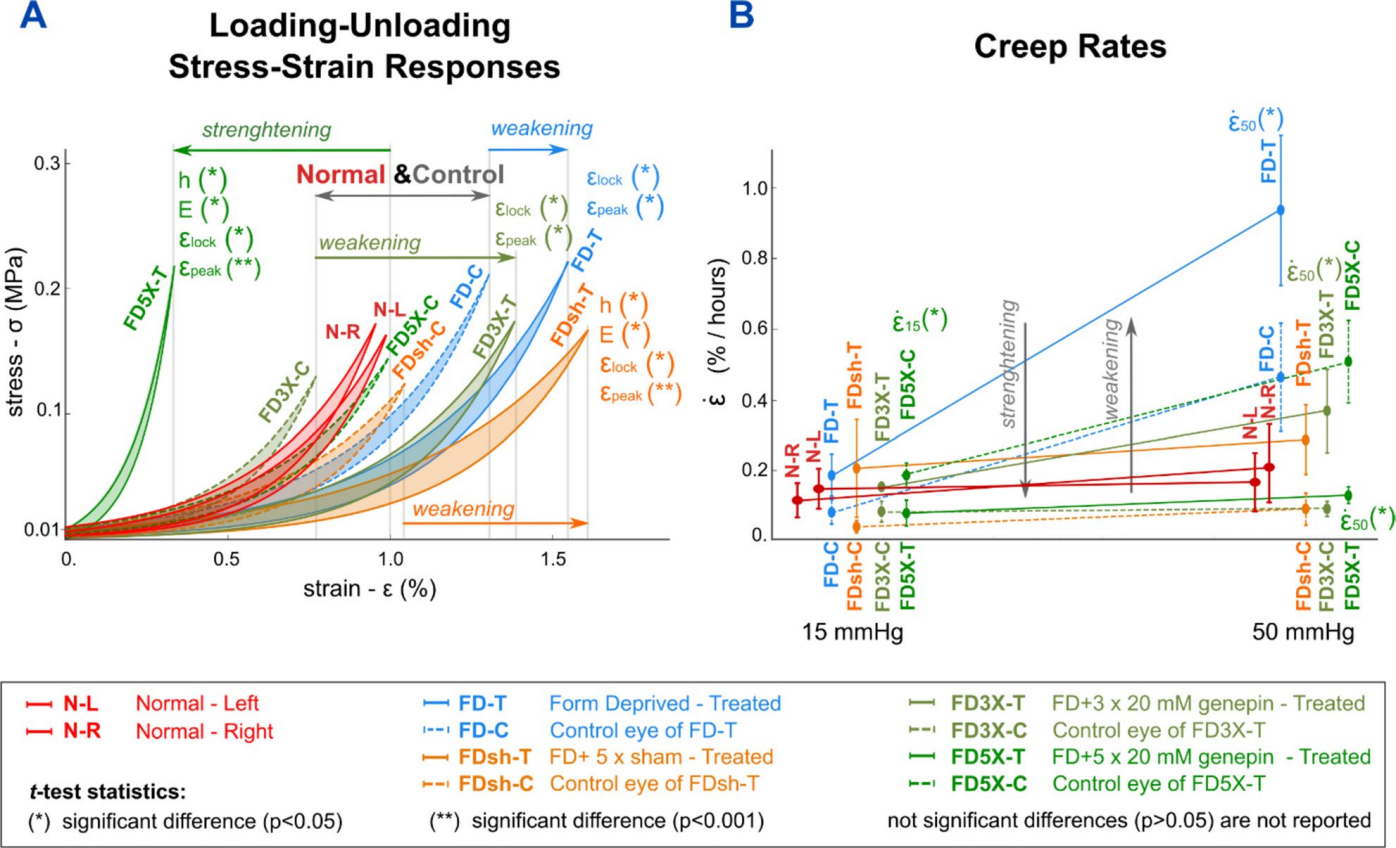
Comparison of the loading–unloading stress–strain responses (**a**) and creep rates at 15 and 50 mmHg (**b**) across Normal (Right and Left), Treated, and Control eye groups. The response of the treated and control eye groups is indicated as Group-T and Group-C, respectively. For the Normal group, we reported the response of the right (N-R) and left (N-L) eyes. Only the mechanical features that showed a significant difference (paired *t*-test) between treated and control eyes are highlighted (*p < 0.05, **p < 0.001).

The average stress-strain responses of the Normal (Right + Left eyes), Treated and Control eyes are plotted in Fig. 2a to illustrate the changes of the mechanical features caused by each treatment. The responses of the Control vs Treated groups were statistically compared and mechanical features (*h*, *E*, *ε*_*lock*_, *ε*_*peak*_) that showed a significant difference (*p*<0.05) were highlighted. From a visual comparison, it is evident that the Control and the Normal groups showed a similar stress-strain response reaching strain levels of approximately 1%. Compared to normal and control eyes, the stress-strain curves of groups FD-T, FD3X-T, and FDsh-T showed a rightward shift toward higher strain values while the FD5X-T showed a leftward shift toward lower strain values. Experimental myopia (FD) induced a significant biomechanical weakening in terms of *ε*_*lock*_ and *ε*_*peak*_ in the FD treated eye, which was also seen in the FD3X group. Surprisingly, the sham injected eyes (FDsh-T) showed additional changes: an increase of the hysteresis *h* and a decrease of the tangent modulus *E*. The decrease of *E* reveals an enhancement of the biomechanical weakening caused by FD in the sham injected eyes, and such a decrease of stiffness occurs along with an increase of *h*. In contrast, 5 genipin injections (FD5X-T) caused a dramatic decrease of *ε*_*lock*_ , *ε*_*peak*_ and *h*, and an increase of the stiffness *E*, demonstrating the biomechanical strengthening of the sclera due to the SXL treatment.

#### Creep rates at 15 mmHg and 50 mmHg

The results of the creep rate tests at 15 and 50 mmHg are shown in Fig. 1h-i and summarized in Table 1. At physiological pressure (15 mmHg) the creep rate difference Δ ${\dot{\varepsilon }}_{15}$ was significantly increased in the pure myopic group (FD) as compared to the Normal group. SXL treatment with 5 genipin injections showed a strong effect resulting in a negative difference of Δ ${\dot{\varepsilon }}_{15}$ that was significantly lower compared to FD. As expected, the creep rate differences at 50 mmHg Δ ${\dot{\varepsilon }}_{50}$ were higher than at 15 mmHg pressure. The FD group showed again the greatest difference in Δ ${\dot{\varepsilon }}_{50}$ (1.108 ± 0.062%). Compared to FD, all three groups with retrobulbar injections (sham and SXL) showed a significant decrease in Δ ${\dot{\varepsilon }}_{50}$. For a graphical comparison, we plotted the creep rate (mean ± SEM) of the Normal group (Right + Left eyes), the Treated (Group-T) and the Control eyes (Group-C) in Fig. 2b. Statistical differences between Treated and Control eyes were also marked. At 15 mmHg, only the FD5X group was found to have a significant difference between treated and control eyes. Increasing pressure to 50 mmHg, the creep rate difference in all groups increased. The creep rate ${\dot{\varepsilon }}_{50}$ was significantly higher in the FD and FD3X groups, but significantly lower in FD5X group. No significant difference of ${\dot{\varepsilon }}_{50}$ was found for the sham group. At both IOP levels, the FD treated eyes were characterized by a higher creep rate compared to the control eye in all groups except for the FD5X. These results suggest that the decreased resistance of the sclera to creep deformations in experimental myopia can be effectively reversed by SXL.

## Discussion

We have provided a comprehensive analysis of the biomechanical changes of the posterior sclera induced by experimental myopia, scleral crosslinking using genipin, and retrobulbar vehicle injections. As expected, form-deprivation induced a significant myopic refractive change, vitreous chamber depth elongation, and scleral thinning. We have found that form-deprivation myopia changed the biomechanical behavior of tree shrew sclera showing a significant increase in *ε*_*lock*_, *ε*_*peak*_, ${\dot{\varepsilon }}_{15}$, and ${\dot{\varepsilon }}_{50}$. The observed increase in the locking strain, peak strain, and creep rates in the myopic eyes is consistent with previous works^6,7,13^. These findings support the notion that scleral remodeling in myopia involves biomechanical weakening of the sclera due to tissue thinning and micro-structural weakening.

The increase in locking and peak strain suggests a biomechanical weakening effect of the myopic sclera that leads to an extended uncrimping response of the internal collagen fiber architecture. A similar weakening mechanism was previously derived from strip testing and inverse modeling^13^. Also, it was observed that cyclic loading amplifies such scleral weakening response in myopic eyes, which can lead to cyclic softening and a continuous increase of the locking strain^12^. Consistent with findings presented by Grytz et al. such a weakening mechanism did not cause significant changes of the elastic modulus at high IOPs (stiffness *E*)^13^. A possible explanation could be that the collagen fiber arrangement when stretched at high loads shows a similar behavior in myopic and normal eyes. No significant differences were found in the hysteresis loop area of the myopic eyes when compared to normal and control eyes, suggesting that the scleral capability to dissipate and absorb mechanical energy remained mostly unchanged after 11 days of form deprivation. However, only one time point was investigated and the involvement of hysteresis and scleral stiffness in the biomechanical weakening of myopic sclera requires further investigations. Our findings are consistent with observed molecular and transcriptomic changes in myopia. Norton et al. reported a reduction of sulfated glycosaminoglycans (GAGs) in the sclera after 21 days of form-deprivation myopia induced in tree shrew eyes^31^. Also, Siegwart and Strang reported a reduction in the mRNA levels of the proteoglycan (PG) aggrecan in myopia^32^. GAGs and PGs are thought to play an important role in scleral biomechanics^33–35^ and their enzymatic degradation can lead to an increase of scleral stiffness and peak stress, and to a decrease of creep rate and hysteresis^33–36^. These mechanical changes may underlay microstructural changes that increase the friction between collagen fibers or alter changes interaction of the collagen fibril network and the hydrated interfibrillar amorphous gelatinous material (ground substance) of PGs and GAGs. It is important that the above mentioned biomechanical changes induced by GAG-removal have been obtained without preconditioning the tissue samples. Interestingly, the biomechanical behavior of preconditioned samples before and after GAG removal was found to be not significantly different^33–36^ It was suggested that preconditioning of scleral samples may cause progressive fiber recruitment, realignment, and straightening of crimped fibers^34^ which may make the biomechanical effects of GAG reduction undetectable. In addition, while most GAGs were artificially digested in the ex vivo experiments reported above, the in vivo GAG and PG changes induced by experimental myopia are likely much smaller, which may explain why no significant changes in *h* and *E* have been found in our experiments. Further biomechanical studies are needed to elucidate the biomechanical role of GAGs and PGs in myopia.

Repeated retrobulbar injections of our vehicle solution composed by 10% of dimethyl sulfoxide (DMSO) and 90% of ophthalmic balanced salt solution (BSS), caused several biomechanical changes including a remarkable increase in the hysteresis loop area. It has to be noted that the eyes were harvested for inflation testing at 35 DVE, which is nine days after the last sham injection on 26 DVE. Consequently, the retrobulbar injection itself or the vehicle solution had a lasting biomechanical effect on the hysteresis of the sclera. While the mechanism is unclear, repeated retrobulbar sham injections may have caused an inflammation response due to the puncture itself. The vehicle solution contained small amounts of DMSO, which is known to increase the permeability of soft tissues^37^. Consequently, the sham injections may have impacted the capacity of the sclera to retain or absorb water through ionic bonding or osmotic effects, which could also explain the slight increase of scleral thickness in the sham group. In addition to the hysteresis, sham injections caused a significant increase in the peak strain differences when compared to the FD group, showing the highest differences among all groups. The locking strain increased as well, but the difference was not significant. The creep rate difference at 50 mmHg was significantly lower in the sham group when compared to the myopic group but not at 15 mmHg. We have also identified a decrease in scleral stiffness when comparing treated with control eyes of the sham group, revealing a biomechanical effect of the vehicle injection. Consistent with the rightward shift of the stress-strain response in Fig. 2a, the increase in peak strain seems to suggest that the sham injections enhanced this biomechanical weakening effect in addition to the effect of myopia alone. Our results suggest that the sham injections caused biomechanical weakening (increased peak strain), but this weakening did not result in accelerated scleral remodeling and axial elongation possibly due to a secondary mechanism. This secondary mechanism may involve altered scleral hydration and/or the increase in the hysteresis as showed previously on scleral porcine strips^38^. Indeed, the sclera hysteresis was highest in the sham injected group in this study. However, this potential sham effect was not significantly different compared to the myopic group and requires further investigation.

The use of genipin for SXL revealed interesting results. 3 genipin injections conducted before inducing myopia were unable to recover the biomechanical weakening effects induced by FD showing significant higher *ε*_*lock*_, *ε*_*peak*_ and ${\dot{\varepsilon }}_{50}$ compared to its control eye (Fig. 2). In contrast, 5 genipin injections, where one injection was performed during myopia progression, resulted in a profound biomechanical strengthening of the sclera. This included a significant increase in the locking and peak strain, and a significant decrease in both creep rates. In addition, 5 genipin injections resulted also in a profound increase in stiffness modulus *E*. The moderate and profound strengthening response is consistent with the moderate (not significant) and profound effect on myopia progression and vitreous chamber depth seen in the FD3X and FD5X group, respectively. It was surprising to us to see that three genipin injections had such a small effect on myopia progression. However, when we include the sham injected group into this comparison, it seems like the genipin injections had to overcome the added weakening effect of the sham injections (increased peak strain) before showing a profound strengthening effect (decreased peak strain). This observation is illustrated by changes seen in the stress–strain response in Fig. 2a showing that the FD’s stress-stain response resulted in a rightward shift (weakening) compared to Normal eyes. This rightward shift increased in the groups with sham injections. 3 genipin injection reversed this trend and caused a left-ward shift (slight strengthening) compared to the sham group, but this shift did not reach beyond the FD group. Only 5 genipin injections resulted in sufficient biomechanical strengthening and a leftward shift of the stress–strain response that went beyond the FD and the normal groups. It is unclear if just the increased number of injections or the additional injection during myopia progression caused the profound strengthening effect in the FD5X group. Furthermore, 5 genipin injections caused a significant reduction in the hysteresis of the sclera when compared to the FD group. This effect may be counterproductive if the previously discussed sham effect that resulted in a reduced vitreous chamber depth was caused by the increased hysteresis.

The limitations of this study mainly concern the generalizability of the results due to the small sample size. However, even with a small number of specimens, we demonstrated that experimental myopia, SXL, and vehicle injections can significantly change the biomechanical behavior of the tree shrew sclera.

A preliminary analysis of our results showed no statistical difference between control and normal eyes for any of the investigated variables. In this case, a control eye can serve as an internal reference for each subject and be used to isolate the treatment effect within-subjects by computing the difference of a response variable between the treated and control eyes. Based on this assessment and to simplify the presentation of our data, results in Table 1 and Fig. 1 are presented as absolute difference between the treated and control eye as commonly done in previous works ^6,9,30^.

Another limitation of our work concerns the global characterization of the sclera’s response by using the average principal strain across the scleral surface. This approach neglects the anisotropic behavior of the tissue that varies locally and regionally across the posterior sclera. In addition, the usage of Laplace’s law (Eq. (2)), widely used in ocular biomechanics to calculate the stress distribution across corneoscleral shells^39^, represents a limitation in our study because it is based on several assumptions that may be violated. While Laplace’s law has been formulated for stress calculation in thin-walled pressure vessels^40^—which is a valid assumption in the case of posterior scleral shells of tree shrews^16^—it assumes that the pressure vessel is a hollow sphere with a uniform thickness. Furthermore, Laplace’s law does not account for complex mechanical properties (anisotropy, viscoelasticity, heterogeneity, nonlinearity) of the tissue. Another limitation of this study is the lack of micro-scale information about changes in scleral structure and composition across the experimental groups, which may have provided mechanistic insight into the observed biomechanical changes. A multi-scale study investigating both, tissue-level biomechanics and micro-structural changes, may be able to clarify the mechanisms that underlay the here reported biomechanical changes.

However, the intent of our study was not to accurately characterize the local stress field of the sclera but to compare the overall mechanical response of the sclera across different experimental groups. In addition, we excluded the analysis of the elastic modulus at low IOPs, because the frame rate of our camera setting (1 Hz) was insufficient to consistently capture the initial linear region at low pressure levels. Moreover, placing the coordinate system at the beginning of each loading stage (loading-unloading cycle) to measure the stress-strain response neglects the effect of strain history (i.e. residual strain) that the tissue underwent during the preconditioning and the 30-min rest period. Finally, the here observed sham effects were unexpected. An additional group with normal visual experience and sham injections would have been useful to fully elucidate the effect of retrobulbar sham injections. Furthermore, potential inflammation effects due to the retrobulbar injections were not investigated. A sham group that includes the needle placement into the retrobulbar space without injecting the vehicle solution would be needed to decipher if the needle placement caused any of the here seen sham effects. Similar to observations made with intravitreal injections^41^ our results stress the importance of including a sham group and the careful evaluation of vehicle injections as they can have profound effects on the experimental outcome variables.

To our best knowledge, this is the first study that in-detail analyzed the viscoelastic changes of the sclera in experimental myopia, after retrobulbar sham injections and SXL treatment using a full-field 3D imaging approach to measure deformations of the scleral shell undergoing IOP inflation. Our results suggest that SXL crosslinking with genipin is effective at strengthening the sclera, but its effective use for myopia control requires particular attention in evaluating the effects on different mechanical features and additional effects caused by the vehicle solution. The analysis of the creep rate alone as previously proposed^6,7^ may be insufficient to characterize the full extent of scleral weakening in myopia. In particular, the surprising sham effect on the hysteresis requires further investigation. While the biomechanical role of the hysteresis on scleral remodeling and myopia progression remains unresolved, it likely impacts all dissipative mechanisms of the extracellular matrix (ECM) of the sclera under dynamic loading (mechanical friction, chemical and electrostatic interactions, physical entanglements of the collagen fibrils). Indeed, IOP is not constant and involves frequent fluctuations^42^. An increased scleral hysteresis as observed in the sham group could effectively dissipate energy caused by IOP fluctuations and reduce the forces that are being transmitted to the ECM, which may reduce collagen sliding and scleral remodeling. Currently, no treatment has been proposed to target scleral hysteresis. Moreover, use of genipin for myopia control remains controversial due to its potential adverse effects on retinal structure and function at high dosages^9^. Our results support the further investigation of SXL agents in general and genipin in particular for myopia control. Based on the observed interaction with our vehicle solution, the strengthening effect of genipin could potentially be enhanced by selecting a vehicle solution that does not induce any biomechanical weakening effect. The exact scleral remodeling and biomechanical weakening mechanisms that lead to axial elongation and myopia remains unclear along with what mechanical pathways should be targeted for effective myopia control. Understanding these points can help to elucidate new and effective treatments that target the sclera. The long-term biomechanical effects of SXL remain unclear and were not the focus of this study. Future work is needed on this topic to optimize SXL as a potential treatment for myopia, including the development of an improved delivery strategy to avoid repeated retrobulbar injections. More research is also required to analyze the effect of vehicle solutions used for SXL, which could expand our understanding of the role of scleral hydration on the viscoelastic behavior of the sclera, as analyzed by Hatami-Marbini and Pachenari^38^, and its effect on scleral remodeling and myopia. Further investigations are required to study the correlation between the refractive error, morphometric measures and the mechanical properties of the sclera as presented by Mc Brien et al.^7^ between axial elongation rate of the eye and scleral creep rate. Moreover, studies are needed to evaluate the effect of dynamic IOP changes (IOP fluctuations^42,43^) on progressive myopia.

## Methods

### Animal model and experimental groups

**Fig. 3:**
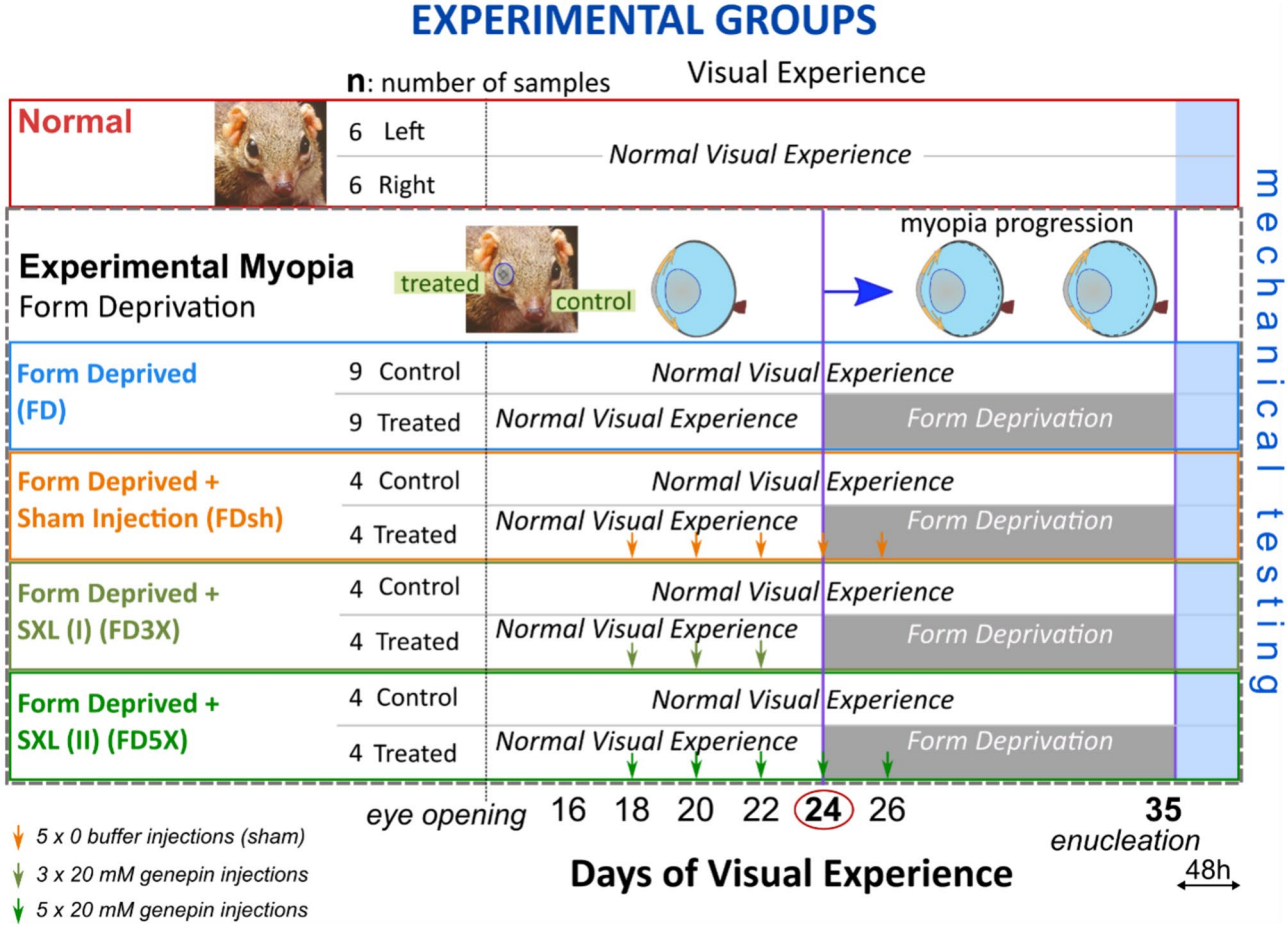
Experimental groups of tree shrews used in this work to test the effects of form deprivation myopia, retrobulbar sham injections, and scleral crosslinking (SXL) using genipin on the biomechanical properties of the posterior sclera.

In this study, we used northern tree shrews (*Tupaia glis belangeri*) that were bred and raised in a colony at the University of Alabama at Birmingham in accordance with The American Association for the Advancement of Laboratory Animal Care guidelines. All studies were performed under an approved Institutional Animal Care and Use Committee (IACUC) protocol at the University of Alabama at Birmingham and conform to the ARRIVE and ARVO guidelines for the use animals in ophthalmic and vision research. Twenty-seven juvenile tree shrews were randomly divided into six experimental groups that differed in *(i)* visual experience (normal visual experience and monocular FD), *(ii)* retrobulbar injections using genipin or vehicle solution (sham), and *(iii)* number of retrobulbar injections (3 or 5 injections). Figure 3 illustrates the different experimental groups. A lightweight aluminum goggle frame with a translucent diffuser covering one eye was installed at 24 DVE to induce progressive FD myopia in one eye while the other eye had unobstructed vision and served as control^6^. At this juvenile age, tree shrews are most susceptible to induced myopia^21^. Retrobulbar injections of vehicle or genipin solution were delivered to the same eye that was exposed to FD as described in detail in our previous work^9^. In short, genipin powder (Wako Chemicals USA, Inc, Fisher Scientific) was dissolved in dimethyl sulfoxide (DMSO; Corning Dimethyl Sulfoxide, Fisher Scientific) and further diluted to a concentration of 20mM. Two groups of animals (n = 4) received either 3 (FDx3) or 5 (FDx5) repeated retrobulbar injections of the genipin solution. One sham group (FDsh, n = 4) received 5 repeated retrobulbar injections (400 µL) of the vehicle solution consisting of 90% of BSS and 10% of DMSO. The injections were performed every other day starting at 18 DVE. Performing injections every other day starting at 18 DVE allowed us to maximize SXL prior to FD treatment. All animals were euthanized at 35 DVE with a lethal injection of xylazine anesthesia. After euthanasia was confirmed, both eyes were enucleated and stored in PBS at 4 °C. A total of 54 eyes have been mechanically tested within 48 h after euthanasia. The animals used in this study represent subgroups of animals that have been used in our previous publications^9,30^, where we investigated the efficacy and safety of SXL using genipin.

### Refractive, vitreous chamber depth, and sclera thickness measurements

Refractive and vitreous chamber depth measurements were performed as described in our previous work^9^. Briefly, we measured refractive error and axial eye dimensions daily in fully awake animals using the Nidek ARK-700A infrared auto-refractor (Marco Ophthalmic, Jacksonville, FL) and Lenstar LS-900 optical biometer (Haag-Streit USA, Mason, OH) following previously established protocols^44,45^. We conducted noncycloplegic refractive measurements^41,44,45^ because cycloplegic drugs may interfere with the emmetropization process and myopia development^45^. Vitreous chamber depth was calculated from the raw Lenstar data using species-specific refractive indices^46^. Optical coherence tomography (OCT) scans of the posterior pole were obtained one day before the animal was euthanized (34 DVE) using the Spectralis OCT2 (Heidelberg Engineering, Inc., Heidelberg, Germany). Scleral thickness was calculated by averaging ten thickness measures, which were manually obtained from one OCT B-scan near the posterior pole as previously described^16^.

### Specimen preparation and mechanical testing

**Fig. 4:**
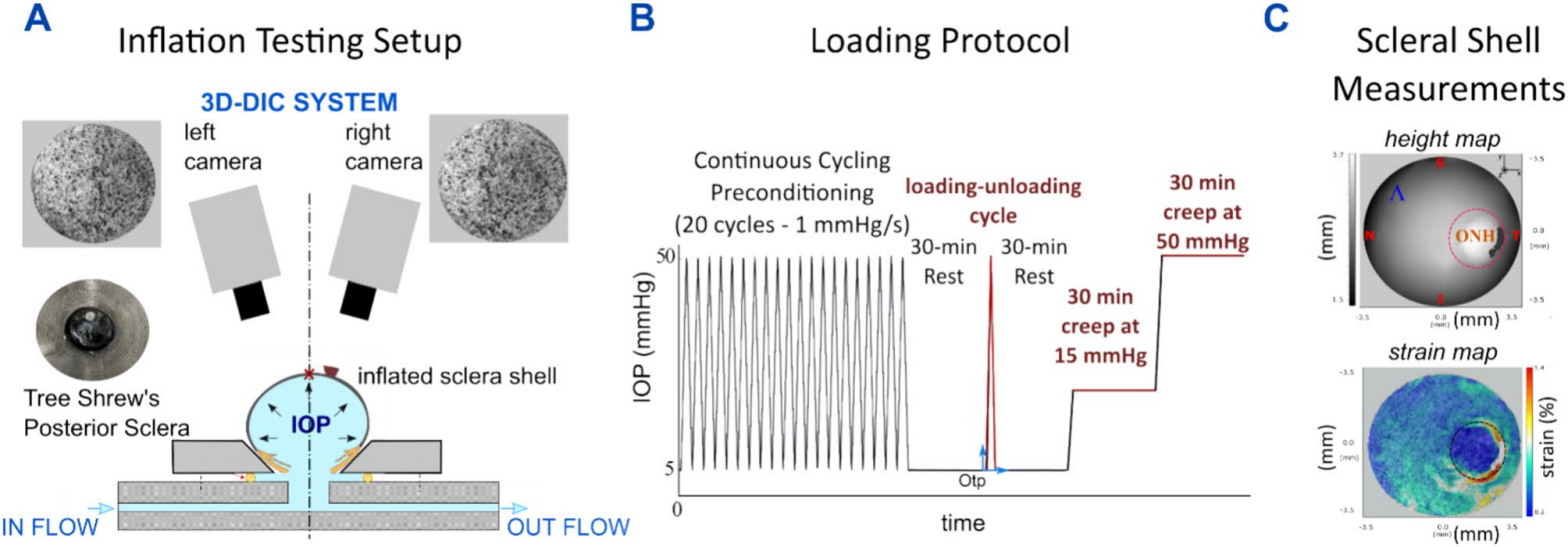
(**a**) Inflation testing setup for measuring tree shrew scleral shell deformations by 3D-DIC. (**b**) Loading protocol consisting of a 20-cycle preconditioning stage, 30-min rest period, a loading–unloading cycle, 30-min rest period, and two creep tests at 15 and 50 mmHg. A coordinate system (Otp) was defined at the beginning of the loading–unloading cycle and used to analyze the viscoelastic stress–strain response. (**c**) Measurements of scleral shell shape represented as height map (top) and average principal strain maps (bottom) computed by LaVision-DaVis software. The optic nerve head (ONH) region shown in the top map was excluded from the analysis.

Specimen preparation and the mechanical testing setup used to analyze the biomechanical response of the tree shrew sclera has been in-detail presented in our previous work^16^. Briefly, each eye sample was glued onto a metal washer and the cornea was trephined through the washer’s hole, the lens and vitreous were removed and the washer mounted on the pressurization chamber allowing the scleral shell to be pressurized by hydrostatic pressure using PBS (Fig. 4a). The baseline pressure was set to 5 mmHg to avoid buckling of the scleral shell while the maximum pressure was set to 50 mmHg to obtain the typical nonlinear J-shaped stress-strain response of collagenous soft tissues. The loading protocol illustrated in Fig. 4b, was composed of a preconditioning loading stage period consisting of 20 loading-unloading cycles by changing the pressure from 5 to 50 mmHg, a rest period of 30 min at 5 mmHg, a single loading-unloading cycle, and two 30 minutes long creep tests at 15 mmHg and 50 mmHg, respectively. Pressure was changed at the rate of 1 mmHg/sec during preconditioning and the loading-unloading cycle. The manometric pressure was measured using a Crystal XP2i pressure gauge (0.1% reading accuracy) that was connected to the pressurization chamber and placed at the same height as the center of the scleral shell. Hydrostatic pressure was applied and controlled by a reservoir mounted on a motorized vertical stage. In order to preserve tissue hydration until inflation testing, the samples were kept in containers with PBS after enucleation. After mounting the scleral shells and coating them with oil, the samples were subjected to baseline IOP (5 mmHg) for 30 min allowing any scleral hydration changes to equilibrate before starting the inflation test.

In addition, during mechanical testing, the hydration of the scleral tissue was maintained *(i)* internally by intraocular PBS in-flow and *(ii)* externally by coating the surface with the mixture of oil and titanium dioxide powder, which prevented the dehydration of the outer surface of the sclera shell due to the humidity gradient from the shell to the lab environment. The use of the oil avoids the need for a humidity chamber as used in other experimental setups (humidity ~90%)^36^. In contrast to oil coating, a humidity chamber could affect the texture-speckled surface during testing due to the formation of micro droplets of water. While it remains unclear if the oil coating had any effect on the biomechanical measurements, this effect should be consistent across groups as all eyes were treated in the same way.

During the loading-unloading cycle and the creep tests, the deformation of the scleral surface was measured by using the three-dimensional Digital Image Correlation (3D-DIC) method. For this purpose, a stereo camera system (The Imaging Source - DMK 23G445 GigE, 1280x960 pixel, monochrome 1/3-inch Sony CCD sensor) was built with a 30° pan angle between cameras, a 10 × 12 mm field of view and ~5mm depth of field. The scleral shell was illuminated by an LED ring placed near the shell to provide dark-field illumination for high-contrast imaging with uniform illumination of the surface. The outer scleral surface was painted with a thin mixture of cosmetic oil and titanium dioxide powder. The mixture served two purposes: the oil prevented tissue dehydration during mechanical testing, and the titanium dioxide enhanced image contrast of the surface. Activated charcoal powder was sprayed on the scleral surface to create a speckled texture required by the 3D-DIC method^47^ (see Fig. 4a). A sequence of stereo-pair images was captured at 1 Hz during the tests. Humidity and room temperature were kept stable at 50% and 22 °C during testing, respectively. The overall acquisition procedure during each testing protocol was controlled by custom code written in Labview (National Instruments, Austin, TX).

### Stress and strain calculation of inflated sclera shells

**Fig. 5:**
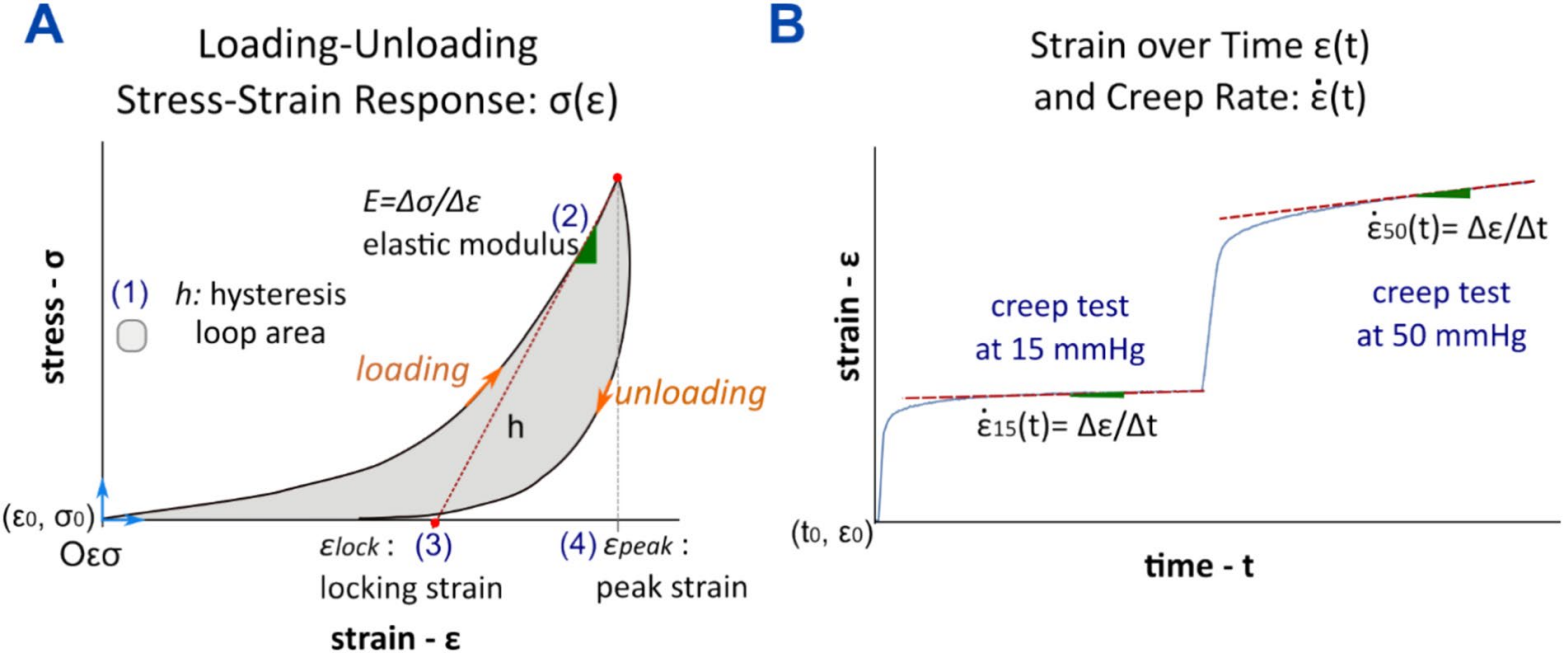
Mechanical features used to analyze the effect of experimental myopia, retrobulbar sham injections, and SXL on the viscoelastic response of the tree shrew sclera. The stress–strain coordinate system (*Oσε*), corresponding to *Otp* in Fig. 4b, was placed at the beginning of the loading–unloading cycle, and used for measuring four mechanical features (**a**): (1) hysteresis loop area *h*, (2) elastic modulus *E* at high IOPs, (3) locking strain *ε*_*lock*_, and *4)* peak strain *ε*_*peak*_. (**b**) The strain over time response of the two creep tests at 15 and 50 mmHg was used to calculate the creep rates ${\dot{\varepsilon }}_{15}$ and ${\dot{\varepsilon }}_{50}$ , respectively.

The sequences of stereo-pair images taken during the inflation tests were processed by LaVision-DaVis software (vs10.0.1, Göttingen, Germany) and used to calculate strain maps of the reconstructed 3D scleral surface, which is shown in Fig. 4c as a height map. The software provided strain maps of the maximum and minimum principal strains (ε_Max_, ε_Min_) on the reconstructed surface. Strain maps calculated at each stereo frame used the first frame of the image sequence as the initial reference for each loading stage studied in this work (Fig. 4b). Because this study is focused on the overall mechanical response of the posterior sclera, we calculated the average principal strain ε_Ave_ across the posterior scleral shell surface ($\Lambda$) excluding the region of the optical nerve head (ONH) (Fig. 5C):

1 $${\upvarepsilon }_{ave} = \frac{1}{\Lambda }\mathop \smallint \limits_{\Lambda }^{{}} \frac{{{\upvarepsilon }_{Max} + \varepsilon_{Min} }}{2}d\Lambda$$

In the rest of this manuscript, we refer to the average scleral surface strain (${\upvarepsilon }_{ave}$) with the symbol ε. The mechanical stress (σ) was estimated by using Laplace’s law for a thin-walled shell^40^, assuming that the scleral shell can be approximated by a spherical vessel with radius (R) and thickness (T):

2 $$\upsigma =\frac{\text{IOP R}}{2 T}$$

The radius of each scleral shell was estimated using CloudCompare software 2.10.2^48^ and the best-fit sphere obtained from the reconstructed surface. The origin of the coordinate system *Oσε* for strain and stress measurements (corresponding to the *Otp*) of the loading-unloading cycle was placed at the beginning of each loading stage as illustrated in Fig. 4B. Hence, scleral strain was assumed to be zero at the origin *Otp* (ε_0_=0), while the initial stress (σ= σ_0_) was calculated using Eq. (2) at the initial pressure of 5 mmHg.

### Characteristic mechanical features of the stress–strain response and creep rate

We analyzed the effect of experimental myopia, retrobulbar sham injections, and different SXL treatments through mechanical features measured during the loading–unloading cycle and the two creep tests (Fig. 4b). In Fig. 5a we depicted a typical loop curve of the scleral stress–strain response σ(ε) during a loading–unloading cycle. Four variables characterizing σ(ε) are indicated: (1) the hysteresis loop area *h* defined as the area between the loading and unloading curves, representing the energy dissipated and/or absorbed by the tissue during one loading–unloading cycle. (2) The elastic or tangent modulus at high IOPs, *E* = Δσ/Δε representing a measurement of the tissue stiffness. *E* was calculated as the slope of the best-fitting line to the stress–strain data of the loading curve for IOPs greater than 35 mmHg^16^. (3) The locking strain *ε*_*lock*_ was calculated as the intersection of the fitted line (used to calculate *E*) with the ε-axis. The locking strain represents the strain level at which scleral collagen fibrils transition from a crimped to a straight arrangement^13,49^. (4) The peak strain *ε*_*peak*_ is the strain at the maximum IOP (50 mmHg), a metric that was previously used to analyze cyclic softening in scleral strips^12^. In addition, as commonly proposed in experimental myopia studies^6,7^, we calculated the creep rate ($\dot{\varepsilon }$(t)) as the slope of the best-fitting line of the strain–time data after a transitory time of 5 min (Fig. 5b). Hysteresis and creep are both characteristic mechanical properties of viscoelastic biological tissues^50^. To plot the average response of each group, the stress–strain data from the loading–unloading cycle was fitted by using the Fung’s exponential function^50^ (*σ (ε)* = *a exp[b ε]* + *c*) for each test. The average response for each experimental group (treated, control, and normal groups) was obtained by averaging the function’s parameters (a, b, c).

### Statistical analysis

Unpaired *t*-test with Bonferroni correction has been used for pairwise group comparisons to assess the effect of experimental myopia, retrobulbar sham injections, and SXL on refractive error, vitreous chamber depth, scleral thickness, and biomechanical properties. Outcome variables were expressed as differences between Right and Left eyes for the Normal group and as differences between Treated and Control eyes for all form-deprived groups. Paired *t*-test with Bonferroni correction were instead used for the comparison between Treated and Control eyes within each treated group (FD, FDh, FD3X, and FD5X). The statistical analysis was performed in RStudio (Version 1.1.463, Boston, MA). Data are plotted as means with error bars indicating the standard error of the mean (SEM). Statistical significance was indicated by *p*-values (*p<0.05; **p<0.001) while not significant differences (*p*>0.05) were indicated by “n.s.”.

## Data Availability

The datasets used and/or analysed during the current study available from the corresponding author on reasonable request.
